# Early Diagnosis of Oral Squamous Cell Carcinoma (OSCC) by *miR-138* and *miR-424-5p* Expression as a Cancer Marker

**DOI:** 10.31557/APJCP.2021.22.7.2185

**Published:** 2021-07

**Authors:** Farzaneh Bolandparva, Mahboobeh Sadat Hashemi Nasab, Abdolreza Mohamadnia, Ata Garajei, Abdollah Farhadi Nasab, Naghmeh Bahrami

**Affiliations:** 1 *Craniomaxillofacial Research Center, School of Dentistry, Tehran University of Medical Sciences, Tehran, Iran. *; 2 *Oral and Maxillofacial Surgery Department, School of Dentistry, Tehran University of Medical Sciences, Tehran, Iran. *; 3 *Chronic Respiratory Diseases Research Center, National Research Institute of Tuberculosis and Lung Diseases (NRITLD), Shahid Beheshti University of Medical Sciences, Tehran, Iran. *; 4 *Department of Biotechnology, School of Advanced Technologies, Shahid Beheshti University of Medical Sciences, Tehran, Iran. *; 5 *Behavioral Sciences Research Center,Shahid Beheshti University of Medical Sciences,Tehran, Iran. *; 6 *Department of Tissue Engineering and Applied Cell Sciences, School of Advanced Technologies in Medicine, Tehran University of Medical Sciences, Tehran, Iran. *

**Keywords:** miR-, 138, miR-424-5p, oral squamous cell carcinoma

## Abstract

**Introduction:**

MicroRNAs (miRs) are a group of endogenous, non-coding, 18–24 nucleotide length single-strand RNAs. These molecules mediate the gene expression and are involved in regulating diverse cellular biological processes, i.e. cell cycle, differentiation, and apoptosis. Aberrant *miR* expression has been shown to be an important event in the pathologies of various types of cancer, including oral squamous cell carcinoma (OSCC).

**Methods:**

Blood samples were obtained from 30 patients (15 cases and 15 controls), to determine* miR-138 *and *miR-424-5p* expression by using real-time PCR and ΔΔCT.

**Results:**

The median CT values of miR-138 were 27.60 and 28.70, while those of miR 424-5p were 29.40 and 30.0 in the case and control groups, respectively. Mann-Whitney test indicated no significant difference in miR-138 and miR-424-5p between the two groups (P > 0.05). However, results obtained by ΔΔCT method showed that *miR-424-5p* expression was 1.96 times higher in the case group, but *miR-138* expression was 3.05 times lower in the plasma of OSCC patients.

**Conclusion:**

Our findings suggest that the evaluation of *miR-138* and *miR-424-5p *expression in serum can be used as potent markers for carcinoma detection and also may be a potentially therapeutic approach in the future. Further longitudinal studies with larger samples are required to verify these findings.

## Introduction

Oral squamous cell carcinoma (OSCC) is one of the life-threatening cancer types worldwide, with a high frequency of recurrence and a low survival rate (Warnakulasuriya, 2009; Siegel et al., 2014). Notwithstanding many efforts made to improve the methods for treating OSCC, the therapeutic efficacy and the five-year survival rate remain unsatisfactory (Haddad and Shin, 2008). Therefore, more understanding of the molecular pathologies underlying OSCC provides a novel insight into the development of effective therapeutic strategies for OSCC. 

In recent years, microRNAs (miRs), a small type of non-coding RNAs, have been found to play a significant role in many diseases, including cancer (Jansson and Lund, 2012; Li and Kowdley, 2012). MiRs are a group of endogenous, non-coding, 18–24 nucleotide length single-strand RNAs that mediate the gene expression. These RNAs are involved in regulating diverse cellular biological processes, including cell cycle, differentiation, and apoptosis. Aberrant* miR *expression has been suggested to be an important event in the pathologies of various types of cancer, particularly OSCC (Wu et al., 2011).

The expression patterns of *miRs* in OSCC represent new directions in the search of oral carcinogenesis (Chamorro Petronacci et al., 2019). Although there are many studies on miRs in serum, plasma, and urine in different types of carcinoma such as OSCC, circulating miRs may play a significant role as diagnostic or prognostic biomarkers in human carcinomas (Singh et al., 2018). These magic molecules can also be responsible for oncogenes in the progression of cancer processes or tumor suppressor genes (Mudduluru et al., 2011; Schwarzenbach et al., 2011). 

Emerging studies have shown that miR-138 acts as a tumor suppressor by targeting many genes through proliferation, apoptosis, invasion, and migration and also can sensitize tumors to chemotherapies (Sha et al., 2017). Findings have indicated that miR-138 has ability to inhibit OSCC cell movement and is able to significantly be downregulated in cancer tissues, but not adjacent normal tissues, in OSCC patients (Zhang et al., 2017). Overexpression of *miR-424-5p* significantly induces OSCC cell invasion and migration, demonstrating that miR-424-5p may function as an oncogenic miR and may contribute to the progression of OSCC. Furthermore, the IL-8-induced tumor cell migration and invasion were prevented when miR-424-5p was specifically inhibited by an antagomir (AM424), which indicates that the induction of miR-424-5p is essential for IL-8-induced cellular invasiveness (Peng et al., 2016). SOCS2, suppressor of the cytokine signaling, has been identified as a novel target of *miR-424-5p*, which its overexpression in oral cancer cells can promote the cell migration and invasion through SOCS2 repression (Peng et al., 2016).

In the present study, we analyzed circulating serum *miR-138* and *miR-424-5p* expression in OSCC patients in comparison with healthy control individuals, to identify whether these biomarkers could be diagnostic tools for the effective OSCC detection. Moreover, we investigated the difference in the expression of two miRs in patients with OSCC compared with healthy people, particularly in the early stage.

## Materials and Methods

*Sample collection*

This study included 15 patients with OSCC and 15 healthy controls (Table 1). The blood samples were derived from OSCC patients who referred to Oral and Maxillofacial Surgery Department of Shariati Hospital, Tehran University of Medical Sciences (TUMS), Tehran, Iran. The blood samples of healthy controls were collected from healthy individuals who were hospitalized for the surgical treatments of esthetic or traumatic fracture. The exclusion criteria in this study included OSCC patients with a history of any other malignancies or a history of head and neck radiotherapy or chemotherapy, cases with known immunodeficiency disorder such as AIDS, patients in end-stage or inoperable OSCC, and cases with immune or autoimmune diseases. A written informed consent was received from each patient, and the protocol of the study was approved by the Ethics Committee of TUMS (ethical code: IR.TUMS.DENTISTRY.REC.1398.038).

*Serum samples storage and total RNA extraction*

Peripheral blood samples were obtained before any therapeutic intervention, including surgery or preoperative radiation. All the samples were collected in clot stimulator tube. The basic clinical information was acquired from each participant. The blood was kept for 45 minutes to allow clotting for serum separation and then processed in accordance with the instruction recommended by Mircury Exiqon (Denmark). Thereafter, the blood samples were centrifuged at 2,500 ×g at 4°C for 15 minutes. Subsequently, the fluid was recentrifuged at 4°C at full spin for 15 minutes to remove the remnant contaminants, including erythrocytes. The serum was stored at -70°C until processing for total RNA isolation. As per the manufacturer’s protocol, total RNA was extracted from serum samples using Mircury Exiqon. For this purpose, the miRNeasy Mini Kits and miRNeasy serum (both from Mircury Exiqon) were employed to extract miRs from serum samples. According to the guidelines of manufacture, optical densities for RNA extraction were 260/280 ratio.

*cDNA synthesis*

All the extracted cDNA were normalized to 2 μg, and then the cDNA synthesis was carried out by using cDNA Syntheses Kit (Universal, Exiqon). Synthesized cDNAs from the total RNA stem-loop were used in real-time RT-PCR. Reverse transcriptase (RT) reactions were comprised of the following reagents: 2 μg of RNA sample, 50 nmol/L of stem-loop RT primer, 2× RT buffer, 0.5 mmol/L of each dNTP, and 4 U/μL of M-MLV RT. Reactions (20 μL) were incubated in a PCR system at 37°C for 50 minutes and 85°C for 5 minutes. The samples were then held at 4°C.

*Real-time PCR*

Real-time PCR was performed using SYBR Green PCR Master Mix (Pars Genum, Iran) on a real-time PCR instrument, consistent with manufacturer’s instruction. Each reaction was accomplished in a volume of 20 μL, containing 50 ng of cDNA, 2 μL of universal primer, and 10 pmol of each primer, along with 10 μL of 2× QuantiTect SYBR Green PCR Master Mix. The PCR amplification reaction consisted of denaturation at 95°C for 5 seconds, followed by 40 cycles at 62°C for 20 seconds and 72°C for 30 seconds. All reactions were carried out in triplicate. The comparative Ct method was applied to analyze the differences in each group in expression levels. 

*Statistical analysis*

All the statistical analyses were conducted by the aid of SPSS 21.0 Software. To compare the findings of the serum expression level of two miRs, Mann-Whitney test was utilized, and differences with a P-value < 0.05 were considered as statistically significant. Gene Runner software was also employed to design primers. Owing to applying Rest Software, the negative mark denotes the elevated gene expression. 

## Results

In total, 30 participants, including 15 OSCC patients and 15 healthy individuals, were enrolled in this study. The mean ages of the control and case groups were 53.67 ± 9.15 and 55.80 ± 11.19, respectively. In both groups, eight males (53.3%) and seven females (46.7%) participated.

*Real-time RT-PCR results*

**Figure 1:**
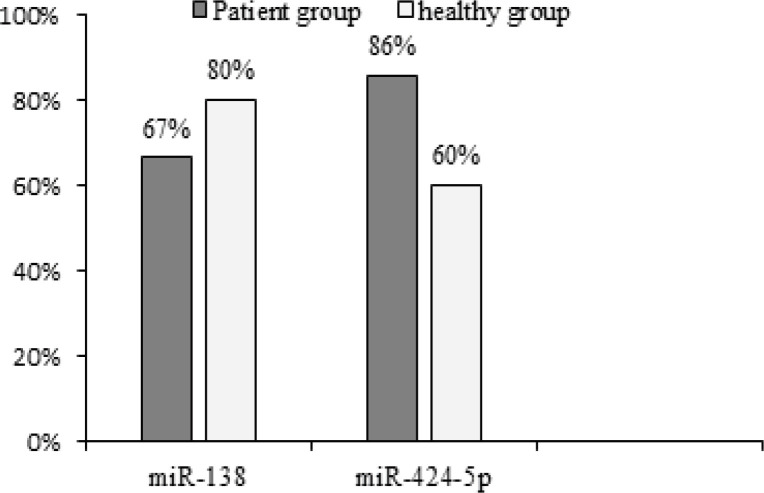
Percentage of Ppositive miR-138 and miR-424-5p in Healthy and Patient Groups

Real-time RT-PCR was performed with the components and methods mentioned in the previous section. From each individual, three constructed cDNA vials were examined for the evaluation of reference genes and markers. The results were interpreted using the above-mentioned method and based on the Melting peak curve. The median CT values of miR-138 were 27.60 and 28.70, but for miR 424-5p, the values were 29.40 and 30.0 in case and control groups, respectively. According to Mann-Whitney test, no significant difference was observed in miR-138 and miR-424-5p between the two groups (P-value > 0.05). The expression of* miR-138* marker was detected in 10 out of 15 OSCC and in 12 out of 15 healthy samples, whereas that of miR-424-5p marker was identified in 13 out of 15 OSCC and in 9 out of 15 healthy samples. Statistical comparison showed a significant difference between the two groups of both *miR-138* and *miR-424-5p* expression, using the Two-sample binomial test (P-value < 0.001; Figure 1). 

*Expression level differences of markers*

**Figure 2:**
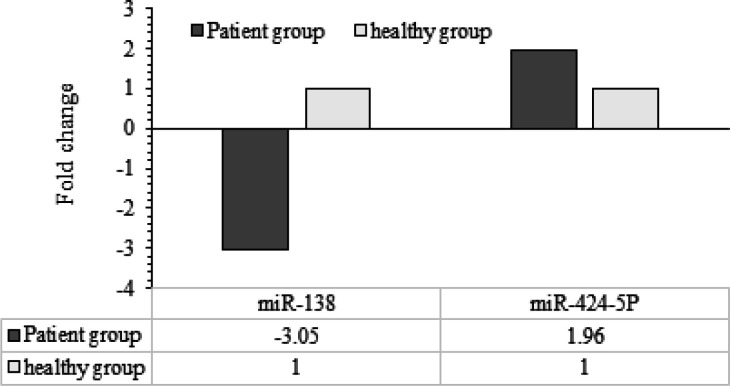
Expression Differences of *miR-138* and *miR-424-5p* in Healthy and Patient Groups

Relative expression levels of markers between healthy individuals and OSCC patients were measured. Results obtained by ΔΔCT method showed that *miR-424-5p* expression was 1.96 times higher in the case group than the healthy one, and* miR-138* expression was 3.05 times lower in the plasma of OSCC patients (Figure 2).

**Table 1:** Demographic Information of All Participants

	Patients with OSCC (N=15)	Healthy individuals (N=15)
Age	55.80 ± 11.19	53.67 ± 9.15
Gender n (%)		
Male	8 (53.3)	8 (53.3)
Female	7 (46.7)	7 (46.7)

## Discussion

Gap between laboratory studies and clinical practice appears to grow up every day, and providing a breakthrough in applying the tumor markers in clinical practice is of high importance. Currently, there are no specific tumor markers for OSCC in the clinical practice; therefore, it is expected that some tumor biomarkers, including novel miRs markers, can be used as potential biomarkers for distinguishing patients with OSCC from healthy individuals (Wang et al., 2018).

MiRs with a length of 18-24 nucleotides are a series of endogenous non-coding RNAs. Their primary function is the regulation of gene expression and RNA silencing in post-transcriptional level (Wu et al., 2011). Previous surveys have reported that the deregulation of miRs is one of the main factors in the initiation and progression of varied cancer types (Shan et al., 2018). In addition, circulating miRs is stably preserved and measurable in blood and thus can serve as reliable biomarkers for the diagnosis or prognosis of a number of carcinomas (Shan et al., 2018; Wang et al., 2018), including OSCC (Singh et al., 2018), and also can suggest a field-effect in oral cancer (Lopes et al., 2018). Zhang et al. have demonstrated that *miR-138* has an inhibitory role in OSCC cell movement, and its expression is significantly downregulated in cancer tissues, but not in adjacent normal tissues in patients with OSCC (Zhang et al., 2017). In Liu et al.’s study, a panel of differentially expressed miRs was identified, i.e. the reduction of miR-138 in highly metastatic cells in head and neck squamous cell carcinoma (HNSCC) cell lines. Ectopic transfection of miR-138 suppressed the cell invasion and led to cell cycle arrest and apoptosis. MiR-138 reduction enhanced cell invasion and suppressed apoptosis. The same authors also suggested that miR-138 acts as a tumor suppresser and could be used as a therapeutic target for HNSCC patients at risk of metastasis (Liu et al., 2009). Yu et al. showed the downregulation of miR-138 in tongue SCC (TSCC) and concluded that miR-138 is responsible for TSCC cell migration and invasion by concurrently targeting Rho-related GTP-binding protein C and ROCK2. These researchers also deduced that miR-138 could be used as a novel therapeutic target for TSCC patients at risk of metastatic disease (Yu and Li, 2016). Peng et al. have emphasized that the overexpression of *miR-424-5p *significantly induces OSCC cell invasion and migration, implying that miR-424-5p may act as an oncogenic miR and may contribute to the progression of OSCC (Peng et al., 2016). According to a meta-analysis result, *miR 424-5p* expression was upregulated in cancerous compared to non-cancerous tissue (Zeljic et al., 2018). In this study, we demonstrated that serum miR-138 and miR-424-5p could be considered as exceptional tumor biomarkers. Based on the results obtained from ΔΔCT method, *miR-138 *expression was downregulated in patients with OSCC (3.05 times), while miR-424-5p indicated upregulation in these patients (1.96 times), which supports previous reported results.

Considering that the overall survival of patients with OSCC is highly poor, screening novel diagnostic biomarkers seems to be an urgent need in confronting with OSCC patients. Blood, in comparison with other body fluids, is generally more applicable as it is readily attained in a non-invasive approachable manner and can be stored for a long time. Thus, since 2008, circulating miRs is known to be diagnostic biomarkers and important approaches for the blood-based detection of human cancer (Mitchell et al., 2008).

The measurement of miRs in serum can assist in the earlier detection of cancers, including OSCC. Circulating miRs might serve as biomarkers for risk of OSCC development and prognosis, as well as response to treatment. Despite recent advances in the field of miRs, therapeutic utility of miRs requires further studies with relying on an precise insight into the real mechanisms of these RNAs in OSCC.

In conclusion, the findings of the present study uncovered that the evaluation of *miR-138* and *miR-424-5p *expression in serum can be used as potent markers for carcinoma detection and also may be a potentially therapeutic approach in the future. More longitudinal studies with larger samples are necessary to confirm these findings.

## Author Contribution Statement

The authors confirm contribution to the paper as follows: study conception and design: F.B, N.B; data collection: M.H, A.GH; analysis and interpretation of results: A.F, F.B, A.M; draft manuscript preparation: N.B, A.M. All authors reviewed the results and approved the final version of the manuscript.
